# Observations on Mr. Maryan's Treatise

**Published:** 1810-07

**Authors:** 


### *To the Editors of the Medical and Physical Journal.*

GENTLEMEN,

PERMIT me to avail myself of the opportunity afforded by your Journal, of making a few remarks upon a pamphlet that has lately fallen in my way, entitled "A Treatise explaining the Impossibility of the Disease termed Hydrophobia being caused by the bite of any rabid Animal, by WILLIAM MARYAN, Surgeon."

The title of the pamphlet induced me, though without much expectation of becoming a convert to the Author's opinions, to open its pages with considerable willingness, in hopes of finding something ingenious and new, in support port of an opinion which some have had the confidence to maintain, notwithstanding the great mass of contrary evidence that stand them in the face, respecting the interesting disease, Hydrophobia. The author's preliminary address still farther stimulated my hopes, where he offers his "Observations to the consideration of Medical Practitioners in particular, and to the Public at large, firmly convinced of their very great importance and utility." He observes too, that Witchcraft, a "*disease*" coeval with Hydrophobia, has, from the increase of learning, been gradually abolished; "and," continues he, "I have no hesitation in declaring it to be my firm belief, that when the disease to which I am now calling the attention of the world, becomes more generally understood, and more fully investigated, it will be found to possess as little foundation as that above alluded to," (the "*disease*" Witchcraft.)

Happy indeed should I have been to have found, in the perusal of this pamphlet, that the author could have proved what he here asserts to be his opinion—that a disease usually considered one of the most horrible that human nature can be afflicted with, and one the least obedient of any to medical skill, is nothing but one of the absurd fantasies of ignorance and barbarism!

Mr. Maryan begins his Treatise by condemning the idea that diseases of animals can be communicated to the human species; and to prove that the introduction of the cow-pox is no contradiction to his opinion, he says that cow-pox is first communicated to the person who dresses a horse with greasy heels, whose hands have the cutis abraded. A little local inflammation is thus produced, "but the *animal economy* does not seem to be more affected by it than by blisters, or any other stimulating application that would excite pain. When the *binary* compound is applied to the teat of the cow, it produces cow-pox; and when this *ternary* compound is conveyed to the human race by inoculation, it secures them from that dreadfully ravaging, and often fatal disease, the small-pox." He continues, "Cows are subject to pustulous sores of their teats, and horses to diseases of their heels; but neither of them, till compounded, are capable of affecting man."

Now, let me examine the depth of Mr. Maryan's reasoning. Unfortunately for his theory, though he does not choose to consider the communication of disease from the horse's heel to the hands of the groom as militating against gainst him, experiments have been made, proving that the human subject, by being inoculated with grease, has not only had a vesicle resembling cow-pox in every respect, produced upon the arm, but has also been effectually secured from small-pox by its effects on the *constitution*. Mr. Bryce, in his last excellent Treatise on Cow-Pox, mentions, that Dr. Loy, of Aislaby, succeeded in many instances; * also, that Dr. Sacco, of Milan, has done the same.† Doctor De Carro, of Vienna; and Dr. Friese, director of vaccination in Silesia, have both inoculated successfully with grease; and now they "inoculate with equine or with vaccine matter indiscriminately, being perfectly convinced of their identity." ‡

Here then we see that experiment has sufficiently proved, that a disease, without having passed through the medium of a *binary* compound, can be *immediately* communicated from an animal to man. Mr. Maryan, however, having asserted the contrary in the last quotation from his work, thus reasons upon it. "As we are not acquainted with any disease (from animals) that, uncompounded, can affect the human species, i. e. solely from the juices of any singly, therefore do I conclude that there can be no possible resemblance between the cow-pox and the bite of a mad dog." Meaning, I presume, that cow-pox is no proof of any morbid animal fluid being communicated immediately from the animal to man; a conclusion which the above experiments completely overturn.

"I never heard (continues he) of a single person, whether coachman, carman, cow-herd, or shepherd, who was *fearful* of becoming affected with any of the diseases of an animal that had died under his care." The fearlessness of these people, allowing it to exist, is no proof of their constitutions being unsusceptible of disease from animals. But as *facts* can generally be opposed to the *opinions* of the author, I beg leave to mention, that Dr. Chisholm has given an account of the Lues Bovina Intertropica, where we are informed, that the animals which died of this complaint, diseased the negroes who handled them. "Touching the flesh in such a manner that part of this sanies adhered to the fingers, produced the same fatal

* Vide Practical Observations on the Inoculation of Cow-pox, &c. by James Bryce, 2d edition.

† London Medical Journal, vol. x. p. 53.

‡ Ibid. vol. xvi, p. 245.

fatal consequences," (viz. pestilential carbuncle with malignant fever.) *

The author having satisfied his own mind, that no morbid animal poison is communicable to man, proceeds next to mention five or six cases of persons who were bitten by dogs, suffering so much mental agitation, as actually to believe they were affected with hydrophobia, though all their symptoms left them, when their minds were made easy on the subject. He dwells particularly upon the case of a Mr. Castleman, of Camberwell, who having been bitten by a dog apparently mad, was so alarmed as to fancy he had the disease, when none existed. He says, p. 24, "The painful sufferings of Mr. Castleman, which he endured uninterruptedly for three or four years, and his great partiality for dogs and sporting, have induced him to pay great attention to the diseases of the canine species, and in no stage of madness in dogs could he perceive they ever refused water, or had any difficulty in swallowing; he declared it therefore to be his firm opinion, that dogs cannot convey any disease to the human race; he is further strengthened in his opinion, by his having been bitten *twenty* times at least by dogs in the same disease, without its ever producing mental or bodily complaints."

Here then terminates the evidence of facts Mr. Maryan has to produce, to prove that hydrophobia is a disease of only a disordered imagination! Because six or eight cases have fallen under his notice of persons, who having been bitten by dogs supposed to be mad, have by the natural horror often excited in the strongest mind by the dread of this most tremendous disease, imagined themselves affected with the symptoms of it, when they really were not; for this reason, forsooth, *there is no such disease as Hydrophobia!"*

It is true, that many persons bitten by mad dogs, indeed the greatest number, are not affected with any disease, the saliva having been wiped off on the clothing through which the teeth often pass before they reach the flesh; some persons being more unsusceptible of this disease than others, as is the case with many in other complaints; the means made use of to destroy the virus on the bitten part, &c. will sufficiently account for the small proportion of those who have been bitten being ill in consequence.

* Vide Edinburgh Medical and Surgical Journal, No, xxi. p. 23.

But how does Mr. Maryan account for the multitude of instances from every quarter, attested by the most respectable authorities, of persons, who, subsequently to the bite of a rabid dog, have been affected with fever, an insuperable dread of swallowing, from the torture former attempts had occasioned, with agonising spasms, delirium, and death? Do all these symptoms arise from the horror excited by the bite of a dog? But in many instances, the patients have never referred their sufferings to this cause, and have altogether forgot their having received a bite. Dr. Clark of Nottingham, relates the case of a child, *seven years and a half old,* who was bitten, and died in the usual dreadful manner.* I beg leave to ask Mr. Maryan, is it from the effect of *imagination* that *horses* after having been bitten, have been violently affected, and died? That this has occurred is sufficiently known and authenticated.† An instance has come to my own knowledge, for the truth of which I can vouch, of the greater part of a flock of sheep having been bitten, becoming ill, and dying in consequence. And are these hysterical sheep, and hypochondriacal horses, so terrified at the idea of being affected with hydrophobia, as to fancy themselves ill, and to die?

One argument of Mr. Maryan's is, dogs do not always dislike water when mad; indeed, from his own experience, and that of his friend, "Mr. Castleman, who had been a *sportsman for these forty years,* dogs never labour under the disease which is attributed to them, but will take *water* in preference to any thing else, to the last hour of their existence," therefore it is impossible they can communicate a disease, *all* the symptoms of which they do not suffer themselves. Now, granting this assertion to be correct, (which, by the way, it is not, according to the experience of many, though it may be so in the practice of Messrs. Castleman and Maryan), what is there so preposterous in the idea, that a disease communicated from an animal to man, should vary in its train of symptoms in the latter, from what appeared in the former? Do we not see that diseases communicated from one human subject to another, frequently run different courses? The mildest distinct

---

* Vide Edinburgh Medical and Surgical Journal, No. xxi. p. 131.

† Vide in London Medical and Physical Journal, vol. xxiii. No. 131, Mr. Surr's account of five horses, which were seized with such severe symptoms in consequence of being bitten, as to die, or to render their being killed necessary.

tinct small-pox in one person, will frequently communicate by infection to another, the most violent and confluent kind. The slightest attack of scarlatina, with little fever, and no affection of the throat, will sometimes appear, when communicated to another, in the form of the most fatal scarlatina anginosa.

When persons bitten by mad dogs are brought to Mr. Maryan, he thus states the practice he adopts. "Whenever I see a person who has been bitten by a mad dog, I inform him it is my belief that no infection can be communicated to him from the dog; but if he should think bathing in the sea, taking the Ormskirk medicine, or any thing that will satisfy his mind; if he can do that, there is no danger, for that the idea of a dog being capable of communicating madness to him, is erroneous; that the disease is only a mental one," &c. Happy would it be for mankind if Mr. Maryan could cure hydrophobia in this way!

To soothe the tortured mind of a person bitten by a mad dog before the disease is come on, by endeavouring to persuade him that he will escape being affected by it, is rational and judicious; but when the symptoms of the disease have appeared, particularly if the patient do not attribute his complaints to the bite, what man in his senses would make use of Mr. Maryan's language!

Upon opening this pamphlet, I fully expected to have found that the author had compared the symptoms of hydrophobia with those of tetanus, and had endeavoured to point out their resemblance in many respects, and that he would have accounted for the hydrophobic symptoms, by supposing them to arise from the effects of the wound alone, and not from any specific virus. Considerable scope for ingenuity is afforded by this view of the subject, and some useful practical remarks might have been deduced from this comparative view of the two diseases; but instead of this, Mr. M. has decided the matter in a simpler manner, by attributing all the symptoms of hydrophobia to hyphochondriasis and melancholia.

It would, Gentlemen, be insulting your readers, were I to aduce any of the numerous cases that have been published in your Journal, or in other books, to show the absurdity of this opinion, or to show that "*exerting our energies,*" will no more cure hydrophobia, than it would stop the bleeding of a wounded carotid!

I leave it to your readers, to judge how far Mr. Maryan has *proved* "the impossibility of the disease termed hydro-

 phobia phobia being caused by the bite of any rabid animal." It is indeed a libel on the word *proof*, to apply it to conclusions drawn from premises so false as Mr. Maryan's are.

It appears to me to be a duty which medical men owe themselves and the public, to prevent the spreading of opinions which are in themselves evidently false, and which may be productive of bad consequences to Society. The circumstance of a book having been written to prove there is no danger in the bite of a mad dog, may be injurious by preventing many from using the proper precaution of confining dogs that appear ill, and of adopting the most approved preventive means, when any one has been bitten.

I am, &c.

CENSOR.

*May*, 1810.

